# Determinants of Food Insecurity and the Choice of Livelihood Strategies: The Case of Abay Chomen District, Oromia Regional State, Ethiopia

**DOI:** 10.1155/2022/1316409

**Published:** 2022-08-09

**Authors:** Bacha Gebissa, Wandu Geremew

**Affiliations:** Department of Agricultural Economics, Faculty of Resource of Management and Economics, Shambu Campus, Wollega University, Nekemte, Ethiopia

## Abstract

Most of the sub-Saharan African countries including Ethiopia were affected by the food insecurity issue. This study aimed to analyze the drivers of food insecurity, the choice of livelihood strategies, and factors that impact the choices of food security strategies in response to food insecurity in Abay Chomen District of Ethiopia's Oromia region, Ethiopia. The result of this study is based on primary data and obtained from 150 randomly chosen sample households and secondary data generated from various sources. As for the technique of data analysis, this study employed descriptive statistics for the food insecurity index, as well as a binary logistic model and a multinomial logit model for the choice of household livelihood techniques. The findings of the survey showed that 51.3% of the households were found to be food-insecure and 48.7% food-safe in the study area. Furthermore, the result indicated that the average calorie consumption of the households surveyed was 2008.54 kcal for each adult equivalent per day, which is below the lowest calorie necessity of 2200 kcal. The estimated logistic model outcome on the drivers of household food insecurity confirmed the oldness of the household leader, larger family holder, and off-farm income affects negatively, while the gender of the household leader, the size of the built-up area, the number of livestock holdings (except oxen), the number of oxen owned, access to credit, the participation in the sale of cattle, and others affect positively. In addition, the multinomial logit model result indicates that the educational status of the household leader, the size of livestock farming, the number of oxen possessed, access to credit, remoteness to the market, and monthly agricultural earning are the main drivers of the choice of livelihood strategies of concern for the food insecurity of households. As a result, this research attempted to produce a result of analysis with a defined scope, although many questions remain unsolved. Future studies should concentrate on presenting fundamental data on the factors that affect food security status and livelihood strategy, the social, political, natural, and environmental aspects, the descriptive information on the shopping habits of people who experience food insecurity, and the key aspects that increase the vulnerability of the rural poor to food insecurity.

## 1. Introduction

It is predicted that around 870 million people worldwide were undernourished (in terms of food energy) between 2010 and 2012. After the 2016 rainy season failed, the government estimated in early 2017 that 5.6 million people lived from February to June [1]. Severe food insecurity affected roughly 1.4 million people in the Democratic Republic of the Congo, Ethiopia, Northern Nigeria, South Sudan, and Yemen in 2021 [2]. Ethiopia is one of the nations that is most heavily impacted by hunger and food shortages. Chronic food insecurity and temporary food insecurity affect a large share of people in the country. The situation of people with chronic food insecurity is becoming increasingly serious. Food security in Ethiopia is closely related to periodic food shortages and starvation in the country that have been linked to recurrent droughts. In 2017, almost 124 million people in 51 countries and territories confronted acute or worse meal insecurity (2). Food availability is a problem for everyone and specifically for developing nations. Food is both a basic need and a human right, as having enough food in terms of quantity and quality is a crucial issue for a wholesome and productive lifestyle, as well as for the sustainable development of a country for all people [3]. As a portion of Africa and developing countries, Ethiopia is one of the countries most severely affected by food insecurity and famine, as a large part of the country's population is affected by both chronic and temporary food insecurity [3]. In Ethiopia, the severity of the food shortage problem varies from one place to another, depending on the country's natural assets and the quantity to which those assets have developed. There are quite a number of things that have created food insecurity problems in Abay Chomen. Low degrees of production in line with farm and land degradation and little technological development within the agricultural sector pose great challenges in seeking to lessen rural poverty and obtain food security within the rural community, such as soil degradation, termite trouble, Dum Nashe and Amarti problem, loss of oxen, incidence of plant and animal sicknesses, terrible soil fertility, weak advisory services, high lack of painting and poor infrastructure, in addition to crop losses before and after the harvest [4]. There is no adequate study that has been done on the subject to date. So, this study was done to fill this information gap by setting the following objectives: to analyze the determinants of food insecurity in the study area and to analyze the factors affecting the choices of livelihood strategies in response to food security in the study area. Thus, the importance of this research can be pointed out from different beneficiaries' points of view: The primary importance of the study is that it is assumed to assist the policy-formulating bodies and decision-makers to give due emphasis to the food insecurity situation of the area in their attempt to save household livelihoods and lives. The secondary importance of this study is that it might use the findings as a guideline to address food (in) security problems. Finally, the study can serve as a reference for further researchers for those who have an interest in the subject matter and study location. It might help them acquire knowledge and skills.

## 2. Analytical Review of the Study

### 2.1. Theoretical Framework of the Study

The central economic sector that is consistently negatively impacted by climate change is agriculture [5]. All parts of the world are impacted by climate change, which leads to significant agitations in natural systems that might be anticipated to have an impact on upland regions' economic systems. Climate change has a detrimental effect on several sectors, including agriculture, groundwater, and diet, soil quality and organic matter, health problems, and poverty [6]. A developing nation is among those in the region most susceptible to extreme climatic events, such as famines and overflows, changes in temperature, and variations in rainfall, which are the main causes of climatic variability. In Pakistan, agriculture is subject to a variety of dangers, especially in places that are prone to flooding, and crop insurance may be a useful strategy for managing risk. Without crop insurance, the nation has long had to deal with the losses caused by natural calamities. Pakistan is the nation that is most vulnerable to severe climatic disasters, such as floods and droughts [5, 6]. Different parts of Ethiopia have been influenced by the problem of climate change, which results in the problem of food insecurity [7–9].

The concept of food security has changed greatly over time, with many researchers, scholars, and organizations refining and broadening its definitions. The word “food security” is dynamic and has expanded over time to embrace new dimensions and levels of examination [10]. With a lengthy history of famines and food shortages that dates back to the 1960s and has left a sizable section of the population food-insecure, Ethiopia is one of the nations most susceptible to famine [7]. According to Ref. [1], since it is difficult to measure food security, it is typically food insecurity that is evaluated, appraised, or studied in order to identify the potential causes of this scenario or potential future causes and to choose the best course of action. Food insecurity, according to the FAO, is a condition in which some people lack access to appropriate supplies of wholesome foods and, as a result, do not eat the food they require to develop normally and live active, healthy lives [1]. Lack of resources, unable to get resources, lack of food (no availability of food), and improper use of resources result in food insecurity, and changes in time result in instability. In order to move from one to the other, a movement is needed. When analyzing food security, one will look at this change and also at the probability that such a change occurs. Susceptibility to food insecurity refers to the full range of factors that place people at risk of becoming food-insecure. The degree of vulnerability of individuals, households, or groups of people is determined by their exposure to the risk factors and their ability to cope with or withstand stressful situations. Food security, as well as poverty, is used to describe people's welfare at the present time. Vulnerability complements this static picture with a dynamic, “forward-looking” perspective that is used to predict how the welfare of individuals and households may change in the future as a consequence of not being able to face adverse events that may happen to them [1]. There are several tiers of food security, from the family to the individual level and from the global, regional, national, and local levels. At various levels, the factors that determine food security are diverse. In other words, food security is seen as a multifaceted phenomenon that includes social problems at all scales—global, regional, national, household, and individual—along with climate change, armed conflict, natural disasters, and social crises. The agriculture industry, groundwater, nutrition, soil quality and organic matter, health conditions, and poverty are all negatively impacted by climate change [5, 11]. Food security can be considered at national, household, and individual levels. At a national level, it is related to the physical existence of food stocks for consumption, be it from own production or from markets. It is related to the availability dimension of food security and is a function of combinations of domestic food stocks, commercial food imports, food aid, and domestic food production, including determinants of each of these factors. On the contrary, household food security is related to the ability to obtain sufficient food of sufficient quality to meet the nutritional requirements of all household members. Household-level food security mainly relies on the economic freedom and purchasing power of household members, which is again related to income distribution in the household [7].

#### 2.1.1. Choice of Livelihood Strategies

Tactics for making a living are actions taken by households to support their way of life. It varies on both the micro- and macro-scales. The various approaches to describing household livelihood strategies that are available in the real world have been reviewed. Rural families create a diverse range of activities and social support abilities known as “livelihood diversification” in their struggle for survival and development in their standards of living and ways of generating a living. The problem of food insecurity, according to Ref. [5], can be remedied through a greater understanding of social and behavioral patterns, as well as through an integrated and comprehensive view of agriculture, climate change, and livelihood processes when assessing vulnerability. According to another study, promoting local enterprises and government financial assistance improves farmers' long-term livelihoods and eliminates absolute poverty. It also shows that poverty alleviation strategies and natural and social capital for long-term survival have a beneficial relationship [6].

### 2.2. Review of Empirical Studies [21]

A study conducted by [8] found that the diverse socioeconomic characteristics of the household have a significant influence on the level of livelihood diversification in Borena pastoralist communities of Oromia regional state, Ethiopia, using a multinomial logit model. The results of this model show that the main factors are the age of the household head, farm input use, extension contact, market access, credit access, and the size of owned cattle. Therefore, household livelihoods are highly diverse, and policymakers need to reflect on the most suitable ways to support this diversity.

According to a study [12] on farmers' risk perception, vulnerability, and adaptation to climate change in rural Pakistan, the study's findings also revealed that participants in the study area faced a variety of challenges in adopting specific adaptation measures to deal with climate variability, including a labor shortage, an unstable land tenure system, a lack of market access, poverty, a lack of governmental support, an inability to access assets, and a lack of assets themselves.

As shown in a study conducted by [13] on the determinants of pastoral household resilience to food insecurity in the Afar region, northeast Ethiopia, pastoralism in Ethiopia is under increasing pressure, caught in a downward spiral of resource depletion and diminishing resilience against shocks and stresses. This article identifies the determinants of pastoral household resilience to food insecurity in Mille, Afar, and Ethiopia. The data analysis consists of principal component analysis and general linear model regression analysis. It finds that the resilience capacity of households in the study area is very weak to shocks from food insecurity. Family size, age, wealth, distance to the market, irrigation access, utilization of soil and water conservation techniques, credit access, and the livestock diversification index significantly explain the variations in the resilience status of households.

Conferring to a study [14] in Kitui County, the most popular coping mechanisms used by farmers were selling animals to buy food, cutting back on daily meals, selling off family assets, and looking for off-farm employment in metropolitan areas. Additionally, there was a statistically significant difference (*P*=0.01) in the farmers' use of nonincome to purchase food, food assistance for asset programs, reliance on relief food, sale of livestock to purchase food, sale of forest products, reduction in the number of meals consumed daily, and the movement of herds between locations within the four agroecological zones. Analysis using a multivariate probit regression model revealed that various socioeconomic factors had diverse effects on the farmers' decision-making.

Another study conducted by [9] on the determinants of rural livelihood diversification strategies among Chewaka resettlers' communities in southwestern Ethiopia showed that agriculture (43.2%), agriculture plus nonfarm (25.5%), agriculture plus off-farm (19.3%), and a combination of agriculture plus nonfarm plus off-farm (12%) activities are the most pertinent livelihood strategies in the study area. It was found that agriculture has a leading contribution to the total households' income (72.5%), followed by nonfarm (20%) and off-farm (7.5%) activities. Multinomial logit model results revealed that land holding size, educational status, livestock holding, gender, age, market distance, credit access, annual income, access to training, and household size were the major determinants of livelihood diversification strategies. Moreover, poor infrastructural development, lack of working capital, the absence of technical support, inadequate skill training, and lack of awareness are constraints to livelihood diversification in the area.

## 3. Methodology

### 3.1. Description of the Study Area

#### 3.1.1. Geographic Location

**Figure 1:**
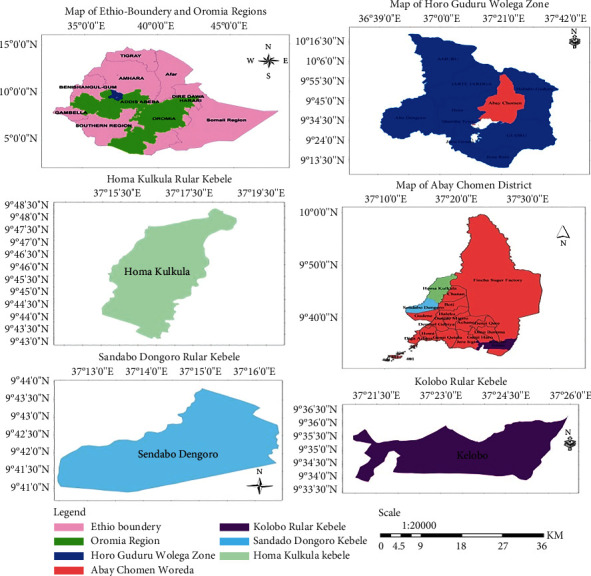
Location map of study area. Source: extracted from the Arc GIS software.

The research was carried out in Abay Chomen District, one of the districts of the Horo Guduru Wollega Zone, Oromia, Ethiopia. It is located about 295.1 km northwest of Addis Ababa, the capital of Ethiopia. Abay Chomen is bordered to the south by Lake Fincha (created when the Fincha Dam flooded the Chomen Swamp), to the southwest by Jimma Ganati and Horo District, to the northwest by Horo Bulk and Jarte Jardaga District, to the southeast by Guduru District, to the north by the Abay River, which separates it from the Amhara region, and to the northeast by Hababo Guduru (Figure 1). The capital of the district is Fincha; other cities are in Migiru and Homi districts of Abay Chomen. The height of this woreda ranges from 880 to 2,400 meters above sea level.

### 3.2. Types, Data Sources, and Data Collection Techniques

This study uses a mixed-methods approach that combines qualitative and quantitative techniques. To comprehend a study problem, the blended approach entails gathering and analyzing several forms of data [15]. The evaluation of household-specific data, such as food security indicators, household composition, asset ownership and access, household income, and food consumption patterns, is made easier with the use of the quantitative method. With the use of a standardized questionnaire, the survey method is used to gather quantifiable data from sample households. Qualitative data on agricultural households' livelihoods and food security are linked to a variety of livelihood security activities, institutional contexts of resource access, susceptibility to shock, subjective importance in relation to food security, experiences, social relationships, and rural livelihood networks.

### 3.3. Sampling and Sample Size Determination

Because there was no document covering all rural homes in Abay Chomen County, multilevel cluster selection was utilized to select a sample. Agroecology was utilized as a stratifying variable since the study of rural livelihoods in Ethiopia is agroecologically sensitive. As a result, this study's sampling method was a stratified area-cluster sample design. As previously mentioned, the procedure began with the classification of the district into three agroecological zones: lowlands, temperate zones, and highlands. Based on data from the Abay Chomen District Agriculture and Natural Resource Bureau, seventeen rural kebeles have been divided into three agroecological zones. According to an unpublished document [4], there are around 3944 households in 3 selected rural kebeles. A lottery process was used to select the study's household sample from the three kebeles' target population. The three criteria were crucial in determining the sample size because they let us gather the information we needed from the sample participants. These include the accuracy, the level of confidence or risk, and the degree of variability in the attributes measured that enable researchers to determine an appropriate sample size [15]. Consequently, with these aspects in mind, the sample size for the collection of data through a questionnaire for this research was determined using the following formula [16]:

(1) $$\begin{array}{c} n=\frac{N}{1+N{\left(e\right)}^{2}}, \end{array}$$

where *n* = the sample size, *N* = the study population, *e* = the level of precision (the acceptable sampling error) (assumed to be 8%), 1 =  the probability of the event occurring at the 95% confidence level.

Accordingly,

(2) $$\begin{array}{c} n=\frac{3944}{1+3944{\left(0.08\right)}^{2}}=150. \end{array}$$

Therefore, for this study 150 sample respondents were selected from those selected kebeles randomly (Table 1).

### 3.4. Method of Data Analysis

Descriptive statistics and econometric data analysis techniques were used in this investigation.

#### 3.4.1. Descriptive Statistics

The data collected from the households in the sample and other sources were analyzed descriptively. Descriptive statistics such as frequency distributions and means were used to describe the characteristics and distribution of strategies for livelihood.

#### 3.4.2. Econometric Model

The factors of food insecurity and livelihood choices among farmers were studied using econometric models. The causes of food insecurity and the choice of livelihood alternatives in the district were investigated using logistic regression models and multinomial logistic regression models, respectively.

*(1) Determinants of Food Insecurity*. For weighted average daily kcal adequacy per AE (adult equivalent), the Federal Democratic Republic of Ethiopia's government has established a minimum demand of 2200 kcal AE (adult equivalent). The threshold is used to differentiate between food-secure and food-insecure households. Food-secure or otherwise food-insecure households are those whose daily intake of kilocalories per AE (adult equivalent) surpasses the subsistence line. The home food security status was the dependent variable, which had two values: 1 for food security and zero for food insecurity. Food insecurity is influenced by a variety of interconnected socioeconomic and climate variables, necessitating multidimensional analysis. The dependent variable, household food security status, is a binary variable. The logit model was used to estimate factors influencing food security status based on the results of the literature review. The cumulative logistic probabilistic model is specified as follows:

(3) $$\begin{array}{c} P_{i}=F\left(Z_{i}\right)=\frac{1}{1+e^{-\left(\alpha +\sum \beta_{i}X_{i}\right)}}, \end{array}$$

where *P*_*i*_ is the probability that an individual *i* is being food-secure given *X*_*i*_ (explanatory variables); *α* and *β* are parameters to be estimated. The logs of odd of the probability that an individual is being food-secure is given by

(4) $$\begin{array}{c} \ln\left(\frac{p_{i}}{1-p_{i}}\right)=Z_{i}=\alpha +\beta_{1}X_{1}+\beta_{2}X_{2}+\ldots +\beta_{k}X_{k}. \end{array}$$

If an error term is assumed, the logit model is expressed as

(5) $$\begin{array}{c} \ln\left(\frac{p_{i}}{1-p_{i}}\right)=Z_{i}=\alpha +\sum_{i=1}^{k}\beta_{i}X_{i}+U_{i}. \end{array}$$

In the case of a dummy dependent variable, OLS is not suitable for estimating the coefficient of the vector of parameters. Therefore, parameters were estimated using maximum-likelihood (ML) techniques. The maximum-likelihood method suggests choosing estimates of the values of the parameters that maximize the likelihood function. In many cases, it is a common practice to maximize the logarithm of the likelihood function itself, and the same results would be obtained.

### 3.5. Operational Definition of Variables with Expected Sign

#### 3.5.1. Dependent Variables

*(1) Status of Household Food Security*. The household's level of food security is a binary dependent variable with values of 1 for those that are secure in their food supply and 0 for those who are not. The grouping of households was caused by the compression between household AE (adult equivalent) per day calorie consumption levels and the average AE (adult equivalent) per day calorie consumption in rural Ethiopia, which was 2100 kcal/AE/day/person [17].

*(2) Choice of Major Livelihood Strategies and Its Determinants*. This study, like most others, developed livelihood plans using income shares from each livelihood activity. The agricultural sector of respondents in the study area had three main sources of income, namely income from agricultural production; income from nonagricultural income-generating activities; and nonagricultural income. These categories of income included a different number of activities: income from agricultural production derived from the activities of staple foods, crops, and livestock; nonagricultural income from small businesses, rural handicrafts, remittance, food/cash, loans, and rent; and farm income derived from recruitment in various forms of employment opportunities such as work and others. For the purposes of this study, the following mutually exclusive combinations of livelihood security strategies were designed for the further analysis of the determinants of livelihood diversification in the study area: The second objective of this study, the choice of major livelihood strategies and factors influencing them, is described by four mutually exclusive livelihood strategies. The dependent variables have the following four nominal outcomes:

  Agriculture only (*y* = 1): This major livelihood strategy included staple crop production, cash crop production, and livestock production.
  Agriculture plus nonfarm (*y* = 2): The combination of agriculture and nonfarm livelihood strategy included agriculture plus petty trades (grain and fruit trade), rural craft, remittance, food/cash aid, loan, small ruminants, and cattle trade.
  Agriculture plus off-farm (*y* = 3): The combination of agriculture and off-farm livelihood strategy included agriculture plus daily labor work, other forms of hire either formal or informal, and natural resource-based activity (firewood collection and charcoaling).
  Agriculture plus nonfarm plus off-farm (*y* = 4): This livelihood strategy combined all activities mentioned above.

Then, the relationships between these four livelihood strategies and socioeconomic variables that deemed to have influence were analyzed using the categorical multinomial regression model. The most widely used categorical multinomial regression models are multinomial logit and multinomial probit models. These two multinomial categorical models have more or less similar results, but in case of multinomial logit model estimation, it is necessary to conduct the Hausman test of independence of irrelevant alternative (IIA) assumption. Hence, the multinomial logit model is preferred for this study. To analyze the determinants of rural household decisions to engage in different livelihood strategies, the assumption is that in a given period, a rational household head is chosen among different mutually exclusive livelihood strategy alternatives that offer the maximum utility. Following [18], suppose for the i^th^ respondent faced with *j* choices, the utility choice *j* is specified as

(6) $$\begin{array}{c} U_{ij}=Z_{ij}\beta +\varepsilon_{ij}. \end{array}$$

If the respondent makes choice *j* in particular, then we assume that *U*_ij_ is the maximum among *j* utilities. So, the statistical model is derived by the probability that choice *j* is made, which is

(7) $$\begin{array}{c} P\left(U_{ij}>U_{ik}\right),\;\text{for all other }K\neq j, \end{array}$$

where *U*_*ij*_ is the utility to the i^th^ respondent from livelihood strategy *j*, and *U*_*ik*_ is the utility to the i^th^ respondent from livelihood strategy *k*.

If the household maximizes its utility defined over income realizations, then the household's choice is simply an optimal allocation of its asset endowment to choose a livelihood that maximizes its utility. Thus, the i^th^ household's decision can therefore be modelled as maximizing the expected utility by choosing the j^th^ livelihood strategy among *J* discrete livelihood strategies.

(8) $$\begin{array}{c} {\text{MAX}}_{j}=E\left(U_{ij}\right)=f_{j}\left(x_{j}\right)+\varepsilon_{ij},\;j=0\ldots j. \end{array}$$

In general, for an outcome variable with *j* categories, let the j^th^ livelihood strategy that the i^th^ household chooses to maximize its utility could take the value 1 if the i^th^ household chooses j^th^ livelihood strategy and 0 otherwise. The probability that a household with characteristics *X* chooses livelihood strategy *j* (*P*_*ij*_ ) is modelled as

(9) $$\begin{array}{c} P_{ij}=\frac{\exp\left(X_{i}^{\prime }B_{j}\right)}{\sum_{j=0}^{J}\exp\left(X_{i}^{\prime }B_{j}\right)},\;j=0. \end{array}$$

with the requirement that ∑_*J*=0_^*J*^*P*_*ij*_=1 for any *i*, where *P*_*ij*_= probability representing the i^th^ respondent's chance of falling into category *j*, *X* = predictors of response probabilities, and *B*_*j*_ = covariate effect specific to j^th^ response category with the first category as the reference.

Appropriate normalization that removes an indeterminacy in the model is to assume that *B*_1_ = 0 (this arises because the sum of probabilities is equal 1, so only *j* parameter vectors are needed to determine the *J* + 1 probabilities) so that exp(*X*_*i*_′*B*_*j*_)=1, implying that the generalized equation (8) is equivalent to

(10a) $$\begin{array}{c} \mathrm{Pr}\left(y_{i}=\frac{j}{X_{i}}\right)=P_{ij}=\frac{\exp\left(X_{i}^{\prime }B_{j}\right)}{1+\sum_{j=0}^{J}\exp\left(X_{i}^{\prime }B_{j}\right)},\;\text{for }j=0,2,\ldots J, \end{array}$$

and

(10b) $$\begin{array}{c} \mathrm{Pr}\left(y_{i}=\frac{1}{X_{i}}\right)=P_{i1}=\frac{1}{1+\sum_{j=0}^{J}\exp\left(X_{i}^{\prime }B_{j}\right)}, \end{array}$$

where *y* = *a* polychromous outcome variable with categories coded from 0, *J*.

The multinomial logistic regression model was used when the outcome of the reliant variable has more than two alternatives for the decision-maker to choose between disordered qualitative or polychrome variables. A multinomial logistic (MNL) model was used to explain the determinants of household livelihood decisions.

#### 3.5.2. Independent Variables (Summarized in Table 2)

Family size is a continuous variable that describes the size of the household's family unit. [8] It is pointed out that increasing (higher) household component tends to exert more pressure on consumption than on the labor it contributes to production. Therefore, this study hypothesizes that a larger household size is expected to affect both food security status and livelihood strategies of households undesirably.

The educational level of the head of household is a continuous variable measured in years of schooling. Education is a form of social capital that can have a positive impact on a household's capacity to make informed production decisions and nutritional status [22]. Based on Ref. [14] and other literature, the higher the educational level of the household head, the more food secure the household is and the more livelihood strategies are expected to be. Thus, this study hypothesizes that advanced years of schooling are expected to affect food security status and the choice of livelihood strategies positively.

Livestock possession (excluding oxen) is a continuous variable measured by the number of tropical livestock units (TLU). Livestock is an important source of food and income for rural households. Households with more livestock produce more milk, milk products, and meat for direct consumption. Besides, livestock enables farm households to have a better chance to earn more income from selling livestock and livestock products, which enables them to increase their purchasing power of stable food during food shortages and could invest in purchasing farm inputs that increase food production and be able to ensure household food security [9]. Thus, this study hypothesizes that owning a greater number of livestock is expected to have a positive effect on food security status and a negative effect on the choice of livelihood strategies of households.

The number of oxen owned is a continuous variable that refers to the number of ploughing oxen. Oxen serve as a source of traction in many developing countries, thereby significantly affecting households' crop production. Animal traction power enables households to cultivate their own land and others' lands through renting, sharecropping, etc., and execute agricultural operations timely [9]. This study hypothesizes that ownership of a larger number of oxen and donkeys is expected to have a positive effect on food security status and a negative effect on the choice of household livelihood strategies.

The frequency of extension contact is a continuous variable that involves a monthly visit by an extension agent. Frequent extension contact enhances households' access to better crop production techniques, improved inputs, and other production incentives, and these help to improve the food security status of households and participate in different livelihood strategies [8]. Thus, this study hypothesizes that the frequency of extension visits is expected to affect food security and the choice of livelihood strategies positively.

Cultivated land size is an incessant variable that refers to the total cropping land cultivated by a household in the past one-year production period. A larger area of cultivated land implies more production and availability of food grains [14]. Higher production and the increased availability of grains produced help in assuring the food security status of households. Hence, the size of cultivated land is expected to have a positive impact on both household food security status and the choice of livelihood strategies.

A dummy variable called “Credit Service” has a value of 1 if farmers have access to credit and 0 otherwise. Credit is readily available, which relieves cash shortages and enables farmers to purchase inputs such as fertilizer, better crop varieties, and irrigation systems. Due to the usage of agricultural inputs, which improve food production and ultimately raise household food security status, farmers who have access to finance would therefore have a favorable impact on crop production. That demonstrates the clear link between loans and the security of household food [9]. Therefore, it is expected that having access to credit will both positively and negatively impact households' levels of food security and their decision-making about means of subsistence.

Off-farm income, measured in birr, is a continuous variable that tracks the total amount of monetary income that any household member earns from jobs or other sources that are not related to farming. [13] As their income improves, they may buy more food to meet their family's needs; a previous research shows that households with nonfarm income sources are less likely to experience food insecurity. Off-farm income is therefore anticipated to have a favorable impact on households' food security status and a negative impact on the tactics they choose to pursue a living.

Monthly farm income is a continuous variable that measures the amount of income obtained from crop production and livestock rearing, measured in birr. [22] Noted that the more the household heads who work in agriculture, the higher their income and the greater their likelihood of having access to food. Household heads with substantial agricultural incomes are more likely to buy a variety of foods to meet their family's food needs. Therefore, it is hypothesized for this study that farm revenue will both positively and negatively impact households' levels of food security and their decision-making over means of subsistence.

The dependency ratio is the ratio of the economically active labor force (those aged between 15 and 65) to the economically inactive labor force (those aged between 15 and 65) [8]. A larger reliance ratio lowers the level of food security in households due to resource limitations as it places a strain on other household members to meet their urgent food needs. The higher dependency ratio also suggests a small labor force, excessive expenditure, and a constraint on household per capita income, all of which affect household members' well-being. It is therefore predicted that the reliance ratio will negatively affect households' levels of food security and their choice of livelihood.

Age in years is a continuous variable that represents the age of the head of the family. The social and physical settings, as well as farming activities, are significantly more richly experienced by older people [14]. In other words, when leaders age, they are expected to have a steady agrarian economy. Additionally, it is projected that older household heads will have better access to land than younger heads since younger males must either wait for land redistribution or share land with their family. Therefore, the projected impact of age on the level of food security in the home and the tactics used to support it could be either good or negative.

A dummy variable, the gender of the head of the family, has a value of 1 if the head of the household is a man and 0 otherwise. According to Ref. [22], women may have a harder time than men getting access to key resources, which helps them increase their productivity and income. They are consequently more likely to experience food insecurity. Therefore, it is anticipated that in this study, gender will be favorably correlated with households' levels of food security and negatively correlated with their selection of livelihood choices.

The market's distance from the closest market centers is expressed in kilometers. Because off-farm employment dictates the income level of rural households, proximity to the nearest market may present a chance for increased income. Additionally, the farmer is more likely to obtain important information and buy agricultural inputs and finished goods needed for family consumption the closer he is to the market [13]. As a result, it is anticipated that this variable will positively impact households' level of food security and the choice of subsistence tactics.

Participation in selling livestock is a dummy variable taking a value of 1 if the farm household participated in selling livestock and 0 otherwise. Households that sell livestock are expected to fare better than their counterparts in terms of food security. This is due to the fact that selling livestock increases their revenue and decreases the risk of food insecurity that households can face. Hence, participation in livestock sales is hypothesized to have an effect on both households' food security status and their choice of livelihood strategies.

A dummy variable called “fertilizer use” has a value of 1 if the farmer applies fertilizer and 0 otherwise. In order to increase farm production, chemical fertilizers such as urea and diammonium phosphate (DAP) are used. Utilizing fertilizer is frequently thought to increase farm yield per square foot [9]. Therefore, it is hypothesized that fertilizer use has a beneficial impact on household food security and a negative impact on the choice of livelihood alternatives.

## 4. Conceptual Framework

**Figure 2:**
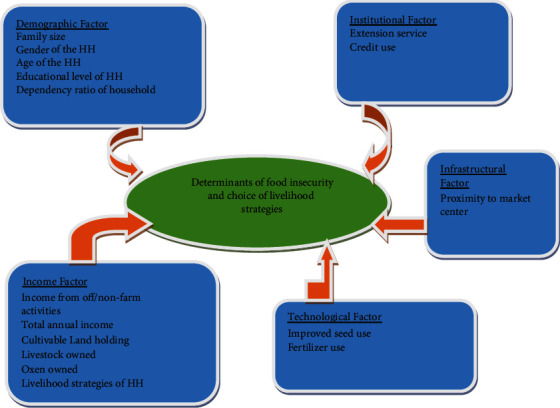
Conceptual framework. Source: developed by the researcher, 2021.

There are five trigger means, such as demographic, institutional, infrastructural, technological, and income factors (Figure 2), by which food insecurity and the choice of livelihood strategies can be determined.

## 5. Results and Discussion

### 5.1. The Food Security Status of the Households in the Study Area

In this study, the distinction between families with and without access to enough food is made based on the number of calories consumed by each adult daily at home. The household's calorie consumption is contrasted with the daily minimum suggested intake of 2100 kcal for adults (the standard calorie intake). The household is categorized as having food insecurity if consumption or intake is below the advised quantity, and as having food safety if it is above the required amount. The food security status of households was measured through a direct consumption survey. The weighted technique was used to gather information on the kind and quantity of food consumed by the household over the course of seven days. Then, the data were transformed into kilocalories and then shared into household mass measured in AE (adult equivalent) and number of days. Then, the amount of energy consumed in kilocalories for the household is compared with the subsistence level per adult and day (i.e., 2100 kcal).

**Figure 3:**
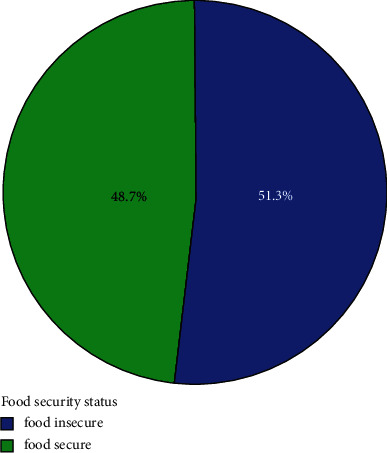
Household's food security status in the study area. Source: Survey Result (2021), *N* = 150.

The result showed that of all households in the sample, 77 (51.3%) households were classified as food-insecure and 73 (48.7%) of them were classified as food-safe (Figure 3). The findings showed that more than half of the households in the study area experienced food insecurity.

This result was deep-rooted by the study made by [3] on the analysis of rural households' food security in western Ethiopia. With regard to the breakdown of the nourishment security status of households according to kebeles, 22 (40.7% of the total sample) of 54 households in Kolobo kebele were food-insecure and 32 (59.5% of the total sample) households were food-safe (Table 3). This shows that there are more households with food security in Kolobo kebele than households with food insecurity. In addition, the study found that of the 50 homes in Homa Kulkula kebele that were surveyed, 23 (or 46% of the total sample) experienced food insecurity, while the other 27 (or 54% of the total sample) experienced food safety (Table 2). In both kebeles, there are more food-secure households than food insecure ones. Furthermore, the research showed that of the 46 families examined in Sandabo Dongoro kebele, 32 (69.6 percent of the total sample) experienced food insecurity, while the other 14 (30.4 percent of the total sample) had access to a supply of food that was safe (Table 3). In this district, too, there are a larger number of food-insecure households than food-secure households.

### 5.2. Livelihood Strategies for Rural Households

**Figure 4:**
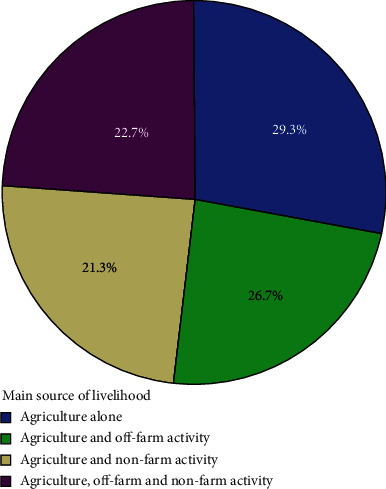
Rural households' livelihood strategies.

Farmers in the study area have used a variety of approaches to secure their livelihoods. A breakdown of the various livelihood security techniques used by households in the research area is provided below. Based on the analysis of the activity portfolios of households, approximately four different patterns of livelihood security strategies may be identified (Figure 4).

The outcome of the descriptive statistics (pie chart) revealed that farmers in the research area most usually employ agricultural activities alone as a strategy for livelihood. About 29.3% of the sampled families relied solely on agricultural (plant and animal production) activities for their means of subsistence. Additionally, roughly 26.7 percent of households relied on both farming and nonfarming occupations for a living. To make a living, farmers in the study region combined farming and raising cattle with off-farm pursuits including beekeeping, raising chickens, and working on other farms. On the contrary, agricultural and nonagricultural activities were used as a source of income in roughly 21.3 percent of the rural households in the research area. To make a living, they combined farming and ranching with nonagricultural pursuits including sporadic work and small-scale trading. Finally, in order to support their families, about 22.7% of the sample's households engaged in a mix of agriculture, nonfarm, and off-farm activities.

### 5.3. The Determinants of Rural Households' Food Insecurity Status in the Study Area

To investigate the factors influencing rural families' food security status, a binary logistic model was applied. The model was chosen based on the rationale explained in the Methodology section above. Results of the binary logit model of the determinants of food security in households using data from a cross-sectional survey of 150 sample households are shown in Table 4. The likelihood-ratio test from the model result showed that the overall model is significant with 1%(*P* < 0.001). The result of the model estimation also showed that 9 of the 14 explanatory variables have a significant influence on the food security of households.

As a result, only the household head's age, gender, family size, amount of cultivated land, number of livestock holdings, number of oxen owned, access to credit, involvement in selling livestock, and off-farm income were statistically significant factors in determining a household's food security status (Table 4). Therefore, only variables with statistically significant coefficients were considered in this study.

#### 5.3.1. Age of the Household Head

With a probability of 5%, this variable has a negative and significant impact on the food security status of households in the research area. The odds ratio for the head of the household revealed from the model's results that the food safety was reduced by a factor of 0.0489 for every year that the household head's age increased. This suggests that older household heads are more likely than younger ones to experience food insecurity. This is because older household heads are less productive and lack the confidence to manage larger farms than their younger counterparts. In addition, older households could not participate in other income-generating activities. On the contrary, older households have large numbers of families and their resources have been distributed among their members. This result is in line with the findings of Refs. [22, 23].

#### 5.3.2. Gender of the Household Head

In line with the expectations of the study, it was discovered to have a positive and significant impact on the food security status of households at a level of significance of 5%. From the model result, the odds ratio of the variables showed that households with male bosses increase the households' food safety by a factor of 1.5912. This implies that households with male management are more likely to be food-safe than households with female management. This is due to the fact that mostly male-headed households have better access to different types of resources, which gives them the opportunity to buy and consume the products they want. This result is in conformity with the findings of Ref. [24].

#### 5.3.3. Family Size

As expected, the family size of the heads of households was found to have a negative impact on the food security status of households at a level of significance of 1%. As indicated in the table above, the odds ratio of the family size showed that an additional person in the household reduces food safety by a factor of 0.3499. This suggests that a larger family size compared with a smaller family size in the study area tends to indicate food insecurity. This is because households in rural areas with large family sizes, composed mostly of nonproductive members, could struggle to maintain food security, and, ultimately, due to the high stress on the active workforce and the lower availability of food to each person in the household end up struggling to achieve food security [25].

#### 5.3.4. Cultivated Land Size

The variable was discovered to be associated with the household's level of food security and to have a significance level of 10% when it came to influencing the dependent variable. According to the model output's chance ratio for acreage size, a household's food security is increased by 0.0672 for every additional hectare of land. Greater output and availability of food grains are implied by a greater cultivated area [26]. Increased availability of the produced grain and greater production both contribute to family food security.

#### 5.3.5. Livestock Ownership (excluding Oxen)

At a level of significance of 1%, it was discovered that raising farm animals is positively and significantly associated with households' food security status. The model's output revealed that the odds ratio of livestock ownership increased by a factor of 0.2778 for every additional unit of tropical livestock in the household. For rural households, livestock is a significant source of food and money. For immediate consumption, households with more cattle produce more milk, dairy products, and meat. Additionally, livestock farming provides farm households with better chances to increase their income from the sale of livestock and livestock products, allowing them to increase their ability to purchase stable food during food shortages and to invest in the purchase of agricultural inputs that boost food production and can ensure household food security [27].

#### 5.3.6. Number of Oxen Owned

As expected, the number of ox owners was found to be positive and statistically significant, with a significance level of 5%. From the model output, the odds ratio of the variables showed that having an additional number of oxen in the household increases food security by a factor of 0.3745. Oxen are used as a source of energy and thus have a considerable negative impact on household crop production in many developing countries. The animal traction enables households to farm their own land and others' lands through renting, sharecropping, etc., and carry out agricultural activities on time [26].

#### 5.3.7. Access to Credit

As expected, this variable has a positive and significant influence on the food security status of households at a significance level of 1%. From the model result, the odds ratio of the variables shows that access to credit increases household food security by a factor of 1.7217. This implies that households that have had access to credit services are more likely to have a safe diet than households without access. This is because lending enables households to participate in income-generating activities, so the income generated increases the household's financial standing and purchasing power to avoid the risk of food insecurity. Additionally, it helps to smooth out consumption when the household is faced with temporary food problems. This result is confirmed by the results of Ref. [22].

#### 5.3.8. Selling Livestock

It was found that the sale of livestock has a positive and significant impact on the food security status of households at a level of significance of 1%. From the output of the binary logit model, the odds ratio of the variables shows that selling livestock increases the probability of household food security by a factor of 1.5609. The result implies that households that sell livestock have a greater chance of food safety than their counterparts. This is because selling livestock increases their income and reduces the risk of food insecurity for households [28].

#### 5.3.9. Off-Farm Income

Contrary to predictions, it was discovered that this variable, at a level of significance of 10%, had a negative and significant impact on the food security status of households. The odds ratio supported the binary logit model's finding that a one birr increase in nonagricultural household income lowers food safety by a factor of 1.5896. This shows that households in the study area with low earnings are more likely to have food security than households with greater nonagricultural incomes. This outcome is consistent with that of Ref. [13].

### 5.4. The Study Area's Rural Households' Livelihood Strategies' Determinants

The factors that influence a rural household's decision about its mode of subsistence were determined using the multinomial logistic model. The model study analyzed the other options as alternatives to this option and simply depended on agriculture (agricultural) as the basic category for no diversification (Table 5). The overall model is significant at 1%. For this reason, only those variables were discussed in this study whose coefficients were statistically significant with a probability of less than or equal to 10%. Head of household education, livestock farming, number of oxen owned, access to credit, distance to market, and monthly farm income were important variables in determining household livelihood choices (Table 5). Yet, the rest were insignificant variables.

#### 5.4.1. Education Level of the Household Head

The level of education of the household head had a positive and significant influence on the use of agriculture (on-farm), nonagricultural and agricultural (on-farm), and nonfarm strategies to secure a livelihood with a probability of 5% and 10%. That is, if all other factors remain the same, every extra year of education raises the likelihood of employing techniques for a living that combine farming and nonfarming by 2.32 percent and 1 percent, respectively, and 37 percent as compared to just the most fundamental sector of agriculture (on-farm) (Table 5). This is the fact that education increases the ability of farm households to employ various livelihood strategies [22].

#### 5.4.2. Access to Credit

It was found that this variable has a positive and significant influence on the choice of household livelihood strategies for securing a livelihood in and outside the company with a probability of 5%. A household's likelihood of engaging in agricultural and nonfarm activities has improved by 3.45 percent, according to the model's results, while access to credit has increased by 25.79 birr, all other parameters being held constant (Table 5). This is due to the fact that households with access to credit can easily cover their consumption and other family needs and also rely on needs-oriented livelihoods. In this way, they can easily overcome financial constraints to engage in alternative nonagricultural activities [9].

#### 5.4.3. Livestock Holding

At a significance level of 10%, it had a favorable and substantial impact on the usage of a combination of on-farm, off-farm, and nonfarm livelihood methods. Ceteris paribus, this implies that a 1 TLU increase in animal husbandry improves the likelihood of using a strategy for the agricultural operation + external plus nonagricultural operations as the basis of life by 23.69 percent compared with the benchmark alternative simply on the farm (Table 5). This is explained by the fact that a farmer's wealth position can be approximated by herd size. Farmers with big herd sizes can easily provide for the food and other needs of their families, and they have a better chance of earning more money to put into off- and off-farm income-generating ventures with the aim of accumulating assets for the future [9].

#### 5.4.4. Number of Oxen Owned

It was discovered that the number of oxen owned, at a level of significance of 5%, had a favorable and significant impact on the combined use of livelihood methods such as on-farm, off-farm, and nonfarm. The outcome reveals that, when all other factors are held constant, an increase in the number of oxen possessed by 1 ox decreased the likelihood of relying solely on farming for a living by 29.09 percent compared with the base category-only farm yard.

#### 5.4.5. Distance to the Market

The variable has a negative and significant impact on households' decisions in favor of the diversification approach for agricultural and nonagricultural livelihoods, with a likelihood of less than 1%. According to the model's marginal effect, ceteris paribus, there has been a 173.84% decline in the likelihood that a household will utilize an agricultural (on-farm) plus nonfarm strategy. This is due to the fact that households located far from the market center lack access to knowledge about engaging in nonagricultural activities, which has led to a fall in livelihood and livelihood strategies [13].

#### 5.4.6. Monthly Farm Income

As expected, this variable has a positive and significant influence on households' choice between farms (agriculture only) plus off-farm, farm plus nonfarm, and a combination of farm, off-farm, and nonfarm diversification strategies for agricultural livelihood with a probability of less than 5%, 5%, and 10%, respectively. With otherwise constant factors, the result of the model results in the marginal effect that the probability that a household will be used in-house plus outside, in-house and outside of the company, and a combination of in-house, off-farm, and nonagricultural activities. The activities increased by 0.001%, 0.001, or 0.0005% among farm households whose monthly income increased by 0.02 birr (Table 4). This is so that households with high total monthly incomes may easily cover their consumption demands and other family needs while also relying on results based on their needs for livelihood (such as wealth accumulation, more income). By doing this, individuals can easily get over financial limitations and indulge in pursuits other than farming. This finding is consistent with that found by Ref. [22].

## 6. Conclusions and Policy Implications

Poverty and food insecurity are significant issues that the majority of Ethiopians currently face. Both chronic and temporary (seasonal) food insecurity are acute across the nation. According to several research papers, improving the lives of the rural poor will be crucial in reducing the occurrence. Therefore, every endeavor to improve the lives of the rural poor and their livelihood strategies must include an assessment of the food security status, the livelihood strategies, and their determinants by taking into account food security dimensions at the household level. In order to examine rural households' levels of food security, their means of subsistence, and the factors that influence their decision to avoid food insecurity, researchers looked at Abay Chomen District.

Data from 150 sampled household heads who were interviewed on a set schedule were used in the study. In order to shed light on the many socioeconomic features of farmers, their level of food security, and the differences between households with and without food security, descriptive statistics (mean, percentage, and frequency) were utilized. The study also used a binary logit model and a multinomial logistic regression model to examine household livelihood strategies, food security status, and their respective factors.

The study's findings indicated that, in the study region, 51.3% of the tested households experienced food insecurity, while 48.7% did not. This suggests that there was a food insecurity problem in the research area for more than half of the examined households. The report also showed that the sampled families' average daily calorie consumption was 2008.54 kcal per adult equivalent, which is less than the recommended minimum of 2100 kcal. The report also showed that the range of calorie intake was between 4186.98 and 697.69.

To investigate the factors influencing the level of food security in rural families, a binary logistic model was estimated. The model's findings supported the notion that a household's level of food security was significantly influenced by the head of the household's age, gender, family size, amount of cultivated land, number of livestock holdings, number of oxen owned, access to credit, involvement in livestock sales, and off-farm income. To investigate the households' choice of rural livelihood strategy, a multinomial logistic model was estimated. In light of this, the household's choice of livelihood methods was significantly influenced by the household head's education level, livestock holding, number of oxen possessed, access to finance, distance to market, and monthly farm income.

The coverage of the study area was primarily responsible for this study's limitations. Due to time and money constraints, the investigation was limited to Abay Chomen District. A cross-sectional household survey was conducted because there are not many of the time series or panel data needed to measure food security in the research area. The study could only use cross-sectional data as a result.

Based on the evidence obtained from this finding, there is a need for urgent action aimed at addressing the need for improving the food security status of rural households to enhance their well-being and to reduce the consequences of different shocks in the study area. These may include the following:

Since the gender of the household head is one of the variables that affect rural households' food insecurity status, improving female-headed households' knowledge and access to different livelihood assets to improve the food security status of the rural poor should be prioritized, because families led by women are more impacted by a lack of information than households headed by men.

The creation of awareness for the elderly should be reinforced because the age of the family head affects food security. Therefore, it is important to provide old household heads with capacity building so that precise information may be made available and spread, allowing them to increase production and ensure food security.

As access to credit affected food security status positively, future interventions should focus on improving rural household's access to credit, because access to credit helps rural households to purchase different inputs to improve their production and consumable products and thereby helps them to ensure food security and improve their well-being. Therefore, development partners operating in the study area should implement provision of credit to eligible households using targeting criterion that reflects actual characteristics of households.

As farm income affected the choice of livelihood strategy positively, future interventions should focus on improving farmer's farm income-earning opportunities. Therefore, it is important to emphasize adequate input supply as a policy option for guaranteeing food security because it boosts farm revenue in rural areas.

Even though it is presumed that easier access to markets will result in lower transportation and other market-related transaction costs, study results show the contrary. Therefore, raising farmers' awareness of the value of improved market access on their ability to make informed decisions about the type of output to be produced, the kind of inputs and products to be purchased in the market, etc., aids farmers in improving their current state of food security in the near future.

In general, this study has sought to produce the outcome of the analysis within a given scope, but there are still many questions that need to be addressed. Future researchers must pay close attention to provide fundamental knowledge on the social, political, natural, and environmental dimensions that determine food security status and livelihood strategy choice, descriptive data on food insecure purchasing patterns, and specific traits that make rural poor more vulnerable to food insecurity.

## Figures and Tables

**Table 1:** Sample size determination from selected kebeles.

No	Name of the kebeles	Total sample	Sample proportion	Sample
1	Kolobo	1556	0.39	59
2	H/Kulkula	1145	0.29	44
3	Sandabo	1243	0.32	47
	Total	**3944**	**1**	**150**

Source: study kebele profile.

**Table 2:** Description of variables. Source: own articulation, 2021.

No.	Variable	Type	Measurement	Expected sign
1	Family size	Continuous	Numbers	−
2	Educational level of head of household	Continuous	Numbers	+
3	Livestock ownership (excluding oxen) [19]	Continuous	TLU	±
4	Number of oxen ownership	Continuous	Numbers	±
5	Frequency of extension contact	Continuous	Number per month	+
6	Cultivated land size	Continuous	Hectares	+
7	Credit service	Dummy	No = 0, Yes = 1	−
8	Off-farm income	Continuous	Birr	±
9	Monthly farm income	Continuous	Birr	±
10	Participation in selling livestock [20]	Dummy	Not participate = 0, participate = 1	
11	Dependency ratio	Continuous	Numbers	±
12	Age of head of household	Continuous	Years	±
13	Gender of the head of household	Dummy	Female = 0, male = 1	+
14	Distance to market	Continuous	Kilometers	+
15	Cultivable land holding	Continuous	Hectares	
16	Fertilizer use	Dummy	Not uses = 0, Yes = Uses	

**Table 3:** Household's food security status and its breakdown between districts.

Kebeles	Household's food security status					
	Food insecure		Food secure		Total	
	N	%	N	%	N	%
Kolobo	22	14.67	32	21.33	54	36
Homa Kulkula	23	15.33	27	18	50	33.33
Sandabo Dongoro	32	21.33	14	9.33	46	30.67
Total	**77**	**51.3**	**73**	**48.7**	**150**	**100**

Source: survey result (2021), *N*  = 150.

**Table 4:** Estimates of the binary logit model's parameters for factors affecting the level of food security in rural households.

Explanatory variables	Binary logistic regression result		
	Odds ratio	Std. error.	*P* > |*t*|
Age of the household head	−0.0489^*∗∗*^	0.025	0.047
Gender of the HH head	1.5912^*∗∗*^	0.759	0.036
Education status of the household head	−0.0765	0.071	0.281
Family size	−0.3499^*∗∗∗*^	0.130	0.007
Dependency ratio	0.1941	0.205	0.343
Cultivated land size	0.0672^*∗*^	0.038	0.073
Livestock holding (except oxen)	0.2778^*∗∗∗*^	0.104	0.008
Number of oxen owned	0.3745^*∗∗*^	0.151	0.013
Occurrence of extension contact	−0.1711	0.106	0.105
Access to credit	1.7217^*∗∗∗*^	0.548	0.002
Distance to market	0.2549	0.377	0.499
Sell livestock	1.5609^*∗∗∗*^	0.537	0.004
Access to fertilizer	−0.7681	0.598	0.199
Off-farm income	−1.5896^*∗*^	0.656	0.015
Constant	0.0417	1.689	0.980
Number of observations		150	
Likelihood chi^2^ (15)		63.63	
Log likelihood		−72.04	
Prob > chi^2^		0.001	
Pseudo-*R*^2^		0.3064	

^*∗∗∗*^, ^*∗∗*^, and ^*∗*^ indicate significance at 1%, 5%, and 10% probability levels, respectively. Source: survey result (2021), *N* = 150.

**Table 5:** Parameter estimates of the multinomial logit model for determinants of the choice of livelihood strategies.

Explanatory variables	Livelihood strategies (base category = agriculture alone)								
	Agriculture + off-farm			Agriculture + nonfarm			Agriculture + off-farm + nonfarm		
	Coeff.	Std. er	Margins	Coeff.	Std. er	Margins	Coeff.	Std. er	Margins
Age of the HH head	−0.0159	0.0261	−0.0056	0.0378	0.0314	0.0045	0.0292	0.0302	0.0032
Gender of the HH head	−0.4884	0.6645	−0.1767	0.8503	0.9063	0.0834	1.4601	1.2196	0.1789
Education status	0.1492^*∗∗*^	0.0699	0.0232	0.1418^*∗*^	0.0858	0.0137	−0.1195	0.1036	−0.0248
Family size	−0.0347	0.1329	−0.0125	0.0500	0.1589	0.0043	0.1163	0.1511	0.0146
Dependency ratio	−0.1825	0.2436	−0.0419	0.1489	0.2427	0.0238	0.0953	0.2512	0.0145
Cultivated land	0.0045	0.0219	0.0024	−0.0282	0.0413	−0.0034	−0.0088	0.0344	−0.0005
Livestock (except oxen)	−0.0289	0.1118	−0.0097	−0.0864	0.1378	−0.0159	0.2369^*∗*^	0.1312	0.0337
Number of oxen owned	−0.1688	0.1435	−0.0428	0.0718	0.1412	0.0081	0.2909^*∗∗*^	0.1341	0.0411
Freq. of extension contact	−0.1789	0.1218	−0.0296	−0.0014	0.1395	0.0088	−0.0203	0.1417	0.0041
Access to training	0.3309	0.5115	0.0608	−0.1259	0.6271	−0.0318	0.0366	0.5978	−0.0039
Access to credit	−0.0060	0.1081	−0.0093	0.2579^*∗∗*^	0.1003	0.0345	−0.1019	0.2109	−0.0202
Distance to market	−0.1955	0.6318	0.0743	−1.7384^*∗∗∗*^	0.6548	−0.1808	−0.7128	0.6804	−0.0329
Off-farm income	1.1444	0.8116	0.1786	0.3936	0.7729	−0.0038	−0.0678	0.7096	−0.0624
Monthly farm income	0.0002^*∗∗*^	0.0001	0.00001	0.0002^*∗∗*^	0.0001	0.00001	0.0001^*∗*^	0.0001	0.000005
Constant	−0.0388	1.7479		−3.4828	2.3543		−4.9226^*∗∗*^	2.3815	

Diagnostics: base category: agriculture (on-farm) alone; number of observations: 150; likelihood ratio chi^2^(42): 105.58; log likelihood: −165.96486; Prob > chi^2^: 0.0013; and pseudo-*R*^2^: 0.1845. ^*∗∗∗*^, ^*∗∗*^, and ^*∗*^ indicate significance at 1%, 5%, and 10% probability levels, respectively. Source: survey result (2021), *N* = 150.

## Data Availability

The authors would like to state that they are willing to provide the publisher with the information and datasets utilized for this work.
